# Diethyl­ammonium 4-hy­droxy­benzoate

**DOI:** 10.1107/S1600536810040523

**Published:** 2010-10-23

**Authors:** Yong-Hong Lu

**Affiliations:** a Ordered Matter Science Reserch Center, College of Chemistry and Chemical Engineering, Southeast University, Nanjing 210096, People’s Republic of China

## Abstract

In the crystal structure of the title compound, C_4_H_12_N^+^·C_7_H_5_O_3_^−^, the cations and anions are linked by N—H⋯O and O—H⋯O hydrogen bonds, leading to the formation of a three-dimensional network.

## Related literature

Hydrogen bonds in co-crystals have been widely used to design and synthesize one-, two- and three-dimensional supra­molecular compounds, see: Aakeroÿ *et al.* (2002 ▶). 4-Hy­­droxy­benzoic acid is a good hydrogen bond donor and can form co-crystals with other organic mol­ecules, see: Vishweshwar *et al.* (2003 ▶).
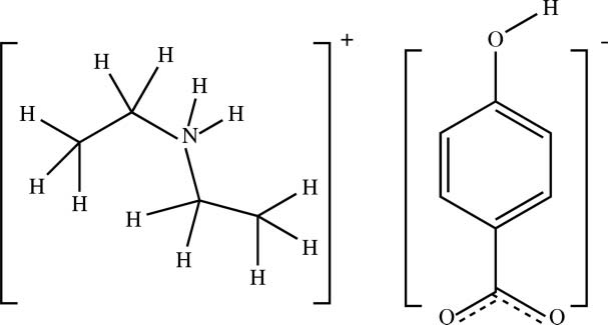


## Experimental

### 

#### Crystal data

C_4_H_12_N^+^·C_7_H_5_O_3_^−^
*M* *_r_* = 211.26
Orthorhombic, 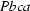

*a* = 12.1270 (13) Å
*b* = 10.6829 (11) Å
*c* = 17.6066 (15) Å
*V* = 2281.0 (4) Å^3^
*Z* = 8
Mo *K*α radiation
μ = 0.09 mm^−1^
*T* = 298 K
0.43 × 0.41 × 0.20 mm

#### Data collection

Rigaku Mercury diffractometer
Absorption correction: multi-scan (*CrystalClear*; Rigaku, 2005 ▶) *T*_min_ = 0.963, *T*_max_ = 0.982
8818 measured reflections
2016 independent reflections
1155 reflections with *I* > 2σ(*I*)
*R*_int_ = 0.048

#### Refinement

*R*[*F* ^2^ > 2σ(*F* ^2^)] = 0.043
*wR*(*F* ^2^) = 0.130
*S* = 1.06
2016 reflections
138 parameters
H-atom parameters constrained
Δρ_max_ = 0.21 e Å^−3^
Δρ_min_ = −0.21 e Å^−3^

### 

Data collection: *CrystalClear* (Rigaku, 2005 ▶); cell refinement: *CrystalClear*; data reduction: *CrystalClear*; program(s) used to solve structure: *SHELXS97* (Sheldrick, 2008 ▶); program(s) used to refine structure: *SHELXL97* (Sheldrick, 2008 ▶); molecular graphics: *SHELXTL* (Sheldrick, 2008 ▶); software used to prepare material for publication: *SHELXTL*.

## Supplementary Material

Crystal structure: contains datablocks global, I. DOI: 10.1107/S1600536810040523/fl2305sup1.cif

Structure factors: contains datablocks I. DOI: 10.1107/S1600536810040523/fl2305Isup2.hkl

Additional supplementary materials:  crystallographic information; 3D view; checkCIF report

## Figures and Tables

**Table 1:** Hydrogen-bond geometry (Å, °)

*D*—H⋯*A*	*D*—H	H⋯*A*	*D*⋯*A*	*D*—H⋯*A*
N1—H1*A*⋯O2^i^	0.90	2.15	2.873 (3)	137
N1—H1*A*⋯O1^i^	0.90	2.16	3.022 (3)	162
N1—H1*B*⋯O2^ii^	0.90	1.83	2.724 (3)	174
O3—H3⋯O1^iii^	0.82	1.82	2.635 (3)	170

Symmetry codes: (i) 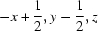; (ii) 
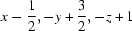
; (iii) 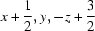.

**Fig. 1.**
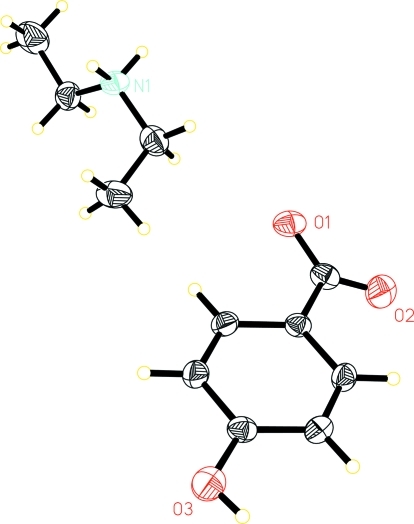
The molecular structure of (I)

**Fig. 2.**
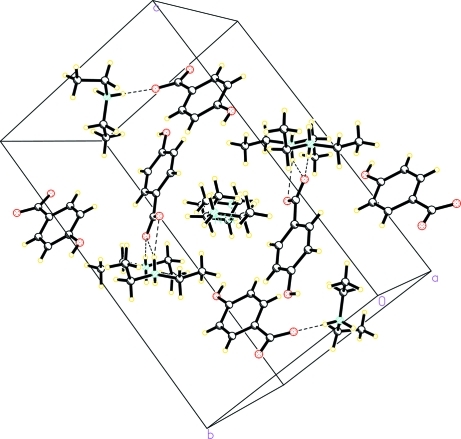
A view of the hydrogen-bonding patterns in (I). Dashed lines indicate hydrgen bonding.

## supplementary crystallographic information

### Comment

In recent years, study of co-crystals has attracted a great many chemists' interest since they can be exploited to improve the physical and/or chemical properties of active pharmaceutical ingredients (APIs). The hydrogen bonds in co-crystals have been widely used to design and synthesize one-, two- and three-dimensional supramolecular compounds (Aakeroÿ *et al.*, 2002).Research into hydrogen bonds experienced a stagnant period in the 1980 s, but re-opened around 1990, and has been in rapid development since then. 4-Hydroxybenzoic acid is a good hydrogen bond donor and can form co-crystals with other organic molecules (Vishweshwar *et al.*, 2003). In this paper, we used 4-Hydroxybenzoic acid and diethylamine to synthesize the co-crystal compound (I).

Compound (I) consists of a diethylamine cation and a 4-hydroxybenzoic acid anion (Fig. 1), therefore, it is a molecular salt. The –NH2 groups of the cations act as hydrogen-bond donors to the O atoms of the carboxyl group of the anions. Moreover, the hydroxyl H atom of the anions also act as hydrogen-bond donors to tone of the O atoms of a neighboring carboxyl group of the 4-hydroxybenzoic acid anions to form a three-dimension network (Fig. 2 and Table 1). One of the H atoms of the –NH2 group links to both O atoms of the –COOH group in an adjacent molecule *via* two N—H···O bonds such that two cations and two anions are linked by hydrogen bonds to form an eight-membered ring.

### Experimental

All reagents were commercially available and of analytical grade. 4- Hydroxybenzoic acid (0.78 mmol, 0.108 g) and diethylamine (0.78 mmol, 0.057 g) were dissolved in ethanol (15 ml). The mixture was stirred for 10 min at room temperature and then filtered. Colorless crystals suitable for data collection were obtained after several days.

### Refinement

The H atoms bonded to C atoms were positioned geometrically and refined as riding, with C—H = 0.93–0.97 Å and *U*_iso_ (H) = 1.2 or 1.5 *U*_eq_ (C) while the H atoms bonded to the N atom and the hydroxy group were located in a difference Fourier map, with N—H = 0.90 Å and O—H = 0.82 Å and then refined with a riding model as was used for the H atoms on the C atoms.

### Crystal data

**Table:**

C_4_H_12_N^+^·C_7_H_5_O_3_^−^	*F*(000) = 912
*M**_r_* = 211.26	*D*_x_ = 1.230 Mg m^−^^3^
Orthorhombic, *P**b**c**a*	Mo *K*α radiation, λ = 0.71073 Å
Hall symbol: -P 2ac 2ab	Cell parameters from 4113 reflections
*a* = 12.1270 (13) Å	θ = 2.4–27.4°
*b* = 10.6829 (11) Å	µ = 0.09 mm^−^^1^
*c* = 17.6066 (15) Å	*T* = 298 K
*V* = 2281.0 (4) Å^3^	Prism, colourless
*Z* = 8	0.43 × 0.41 × 0.20 mm

### Data collection

**Table:**

Rigaku Mercury diffractometer	2016 independent reflections
Radiation source: fine-focus sealed tube	1155 reflections with *I* > 2σ(*I*)
graphite	*R*_int_ = 0.048
ω scans	θ_max_ = 25.0°, θ_min_ = 2.3°
Absorption correction: multi-scan (*CrystalClear*; Rigaku, 2005)	*h* = −7→14
*T*_min_ = 0.963, *T*_max_ = 0.982	*k* = −12→9
8818 measured reflections	*l* = −20→20

### Refinement

**Table:**

Refinement on *F*^2^	Primary atom site location: structure-invariant direct methods
Least-squares matrix: full	Secondary atom site location: difference Fourier map
*R*[*F*^2^ > 2σ(*F*^2^)] = 0.043	Hydrogen site location: inferred from neighbouring sites
*wR*(*F*^2^) = 0.130	H-atom parameters constrained
*S* = 1.06	*w* = 1/[σ^2^(*F*_o_^2^) + (0.0464*P*)^2^ + 0.8922*P*] where *P* = (*F*_o_^2^ + 2*F*_c_^2^)/3
2016 reflections	(Δ/σ)_max_ < 0.001
138 parameters	Δρ_max_ = 0.21 e Å^−^^3^
0 restraints	Δρ_min_ = −0.21 e Å^−^^3^

### Special details

**Table:**

Geometry. All e.s.d.'s (except the e.s.d. in the dihedral angle between two l.s. planes) are estimated using the full covariance matrix. The cell e.s.d.'s are taken into account individually in the estimation of e.s.d.'s in distances, angles and torsion angles; correlations between e.s.d.'s in cell parameters are only used when they are defined by crystal symmetry. An approximate (isotropic) treatment of cell e.s.d.'s is used for estimating e.s.d.'s involving l.s. planes.
Refinement. Refinement of *F*^2^ against ALL reflections. The weighted *R*-factor *wR* and goodness of fit *S* are based on *F*^2^, conventional *R*-factors *R* are based on *F*, with *F* set to zero for negative *F*^2^. The threshold expression of *F*^2^ > σ(*F*^2^) is used only for calculating *R*-factors(gt) *etc*. and is not relevant to the choice of reflections for refinement. *R*-factors based on *F*^2^ are statistically about twice as large as those based on *F*, and *R*- factors based on ALL data will be even larger.

### Fractional atomic coordinates and isotropic or equivalent isotropic displacement parameters (Å^2^)

**Table:**

	*x*	*y*	*z*	*U*_iso_*/*U*_eq_	
N1	0.15290 (17)	0.4229 (2)	0.46227 (12)	0.0500 (6)	
H1A	0.1303	0.3844	0.5050	0.060*	
H1B	0.0985	0.4747	0.4474	0.060*	
O1	0.39118 (14)	0.74534 (19)	0.59243 (10)	0.0538 (5)	
O2	0.49892 (17)	0.9105 (2)	0.58731 (11)	0.0685 (6)	
O3	0.75558 (15)	0.59440 (17)	0.83491 (10)	0.0597 (6)	
H3	0.7969	0.6470	0.8534	0.090*	
C1	0.4739 (2)	0.8063 (3)	0.61463 (14)	0.0449 (7)	
C2	0.54591 (19)	0.7521 (2)	0.67521 (13)	0.0371 (6)	
C3	0.62071 (19)	0.8261 (2)	0.71431 (13)	0.0420 (6)	
H3A	0.6239	0.9113	0.7038	0.050*	
C4	0.69034 (19)	0.7768 (2)	0.76835 (13)	0.0417 (6)	
H4	0.7389	0.8286	0.7944	0.050*	
C5	0.68774 (19)	0.6501 (2)	0.78369 (13)	0.0412 (6)	
C6	0.61259 (19)	0.5748 (2)	0.74597 (14)	0.0451 (7)	
H6	0.6095	0.4896	0.7567	0.054*	
C7	0.54265 (19)	0.6254 (2)	0.69286 (13)	0.0420 (6)	
H7	0.4922	0.5740	0.6683	0.050*	
C8	0.2517 (3)	0.4991 (3)	0.48039 (17)	0.0638 (8)	
H8A	0.2304	0.5662	0.5144	0.077*	
H8B	0.2791	0.5368	0.4340	0.077*	
C9	0.3426 (2)	0.4254 (3)	0.51642 (18)	0.0724 (9)	
H9A	0.3139	0.3792	0.5588	0.109*	
H9B	0.3992	0.4815	0.5337	0.109*	
H9C	0.3730	0.3684	0.4799	0.109*	
C10	0.1689 (2)	0.3275 (3)	0.40308 (15)	0.0575 (8)	
H10A	0.2266	0.2702	0.4187	0.069*	
H10B	0.1924	0.3679	0.3565	0.069*	
C11	0.0657 (3)	0.2559 (3)	0.3885 (2)	0.0845 (11)	
H11A	0.0446	0.2118	0.4338	0.127*	
H11B	0.0780	0.1969	0.3483	0.127*	
H11C	0.0080	0.3126	0.3742	0.127*	

### Atomic displacement parameters (Å^2^)

**Table:**

	*U*^11^	*U*^22^	*U*^33^	*U*^12^	*U*^13^	*U*^23^
N1	0.0416 (13)	0.0629 (15)	0.0456 (12)	0.0035 (11)	0.0090 (10)	0.0040 (11)
O1	0.0427 (11)	0.0719 (13)	0.0467 (11)	−0.0014 (10)	−0.0083 (9)	0.0082 (9)
O2	0.0711 (14)	0.0676 (14)	0.0668 (13)	−0.0052 (11)	−0.0119 (11)	0.0315 (11)
O3	0.0530 (11)	0.0591 (12)	0.0670 (12)	−0.0036 (10)	−0.0217 (10)	0.0170 (10)
C1	0.0431 (16)	0.0561 (18)	0.0356 (14)	0.0077 (14)	0.0067 (12)	0.0044 (13)
C2	0.0336 (13)	0.0426 (15)	0.0352 (13)	0.0021 (12)	0.0047 (11)	0.0047 (11)
C3	0.0465 (15)	0.0369 (15)	0.0426 (14)	−0.0032 (12)	0.0029 (13)	0.0063 (12)
C4	0.0405 (15)	0.0444 (16)	0.0402 (14)	−0.0072 (12)	−0.0015 (12)	0.0011 (12)
C5	0.0359 (14)	0.0486 (16)	0.0390 (13)	0.0030 (12)	0.0003 (12)	0.0070 (12)
C6	0.0424 (15)	0.0390 (15)	0.0539 (16)	−0.0020 (13)	−0.0045 (13)	0.0076 (13)
C7	0.0350 (14)	0.0441 (16)	0.0467 (15)	−0.0049 (12)	−0.0022 (12)	0.0005 (13)
C8	0.067 (2)	0.0595 (19)	0.0652 (19)	−0.0111 (16)	0.0033 (16)	−0.0082 (16)
C9	0.0508 (19)	0.092 (2)	0.074 (2)	−0.0046 (18)	−0.0058 (16)	−0.0123 (19)
C10	0.0637 (19)	0.0506 (18)	0.0584 (17)	0.0002 (15)	0.0068 (15)	−0.0044 (14)
C11	0.076 (2)	0.086 (3)	0.091 (3)	−0.018 (2)	−0.028 (2)	−0.003 (2)

### Geometric parameters (Å, °)

**Table:**

N1—C10	1.470 (3)	C6—C7	1.373 (3)
N1—C8	1.483 (3)	C6—H6	0.9300
N1—H1A	0.9000	C7—H7	0.9300
N1—H1B	0.9000	C8—C9	1.495 (4)
O1—C1	1.259 (3)	C8—H8A	0.9700
O2—C1	1.249 (3)	C8—H8B	0.9700
O3—C5	1.358 (3)	C9—H9A	0.9600
O3—H3	0.8200	C9—H9B	0.9600
C1—C2	1.495 (3)	C9—H9C	0.9600
C2—C3	1.386 (3)	C10—C11	1.489 (4)
C2—C7	1.388 (3)	C10—H10A	0.9700
C3—C4	1.377 (3)	C10—H10B	0.9700
C3—H3A	0.9300	C11—H11A	0.9600
C4—C5	1.381 (3)	C11—H11B	0.9600
C4—H4	0.9300	C11—H11C	0.9600
C5—C6	1.385 (3)		
			
C10—N1—C8	115.2 (2)	C6—C7—H7	119.4
C10—N1—H1A	108.5	C2—C7—H7	119.4
C8—N1—H1A	108.5	N1—C8—C9	113.4 (2)
C10—N1—H1B	108.5	N1—C8—H8A	108.9
C8—N1—H1B	108.5	C9—C8—H8A	108.9
H1A—N1—H1B	107.5	N1—C8—H8B	108.9
C5—O3—H3	109.5	C9—C8—H8B	108.9
O2—C1—O1	122.3 (2)	H8A—C8—H8B	107.7
O2—C1—C2	118.6 (3)	C8—C9—H9A	109.5
O1—C1—C2	119.1 (2)	C8—C9—H9B	109.5
C3—C2—C7	117.6 (2)	H9A—C9—H9B	109.5
C3—C2—C1	121.0 (2)	C8—C9—H9C	109.5
C7—C2—C1	121.4 (2)	H9A—C9—H9C	109.5
C4—C3—C2	121.8 (2)	H9B—C9—H9C	109.5
C4—C3—H3A	119.1	N1—C10—C11	111.6 (2)
C2—C3—H3A	119.1	N1—C10—H10A	109.3
C3—C4—C5	119.7 (2)	C11—C10—H10A	109.3
C3—C4—H4	120.1	N1—C10—H10B	109.3
C5—C4—H4	120.1	C11—C10—H10B	109.3
O3—C5—C4	123.1 (2)	H10A—C10—H10B	108.0
O3—C5—C6	117.6 (2)	C10—C11—H11A	109.5
C4—C5—C6	119.4 (2)	C10—C11—H11B	109.5
C7—C6—C5	120.3 (2)	H11A—C11—H11B	109.5
C7—C6—H6	119.9	C10—C11—H11C	109.5
C5—C6—H6	119.9	H11A—C11—H11C	109.5
C6—C7—C2	121.2 (2)	H11B—C11—H11C	109.5
			
O2—C1—C2—C3	16.5 (3)	C3—C4—C5—C6	−1.9 (4)
O1—C1—C2—C3	−164.2 (2)	O3—C5—C6—C7	−179.2 (2)
O2—C1—C2—C7	−161.6 (2)	C4—C5—C6—C7	1.1 (4)
O1—C1—C2—C7	17.7 (3)	C5—C6—C7—C2	0.5 (4)
C7—C2—C3—C4	0.5 (3)	C3—C2—C7—C6	−1.3 (4)
C1—C2—C3—C4	−177.6 (2)	C1—C2—C7—C6	176.8 (2)
C2—C3—C4—C5	1.1 (4)	C10—N1—C8—C9	−66.5 (3)
C3—C4—C5—O3	178.5 (2)	C8—N1—C10—C11	−179.9 (3)

### Hydrogen-bond geometry (Å, °)

**Table:**

*D*—H···*A*	*D*—H	H···*A*	*D*···*A*	*D*—H···*A*
N1—H1A···O2^i^	0.90	2.15	2.873 (3)	137
N1—H1A···O1^i^	0.90	2.16	3.022 (3)	162
N1—H1B···O2^ii^	0.90	1.83	2.724 (3)	174
O3—H3···O1^iii^	0.82	1.82	2.635 (3)	170

Symmetry codes: (i) −*x*+1/2, *y*−1/2, *z*; (ii) *x*−1/2, −*y*+3/2, −*z*+1; (iii) *x*+1/2, *y*, −*z*+3/2.
